# Dysmenorrhea and related disorders

**DOI:** 10.12688/f1000research.11682.1

**Published:** 2017-09-05

**Authors:** Mariagiulia Bernardi, Lucia Lazzeri, Federica Perelli, Fernando M. Reis, Felice Petraglia

**Affiliations:** 1 Department of Molecular and Developmental Medicine, Obstetrics and Gynecological Clinic, University of Siena, Siena, Italy; 2 Department of Experimental, Clinical and Biomedical Sciences, Obstetrics and Gynaecology, University of Florence, Florence, Italy; 3 Department of Obstetrics and Gynecology, Universidade Federal de Minas Gerais, Belo Horizonte, Brazil

**Keywords:** Dysmenorrhea, endometriosis, adenomyosis, menstrual disorders

## Abstract

Dysmenorrhea is a common symptom secondary to various gynecological disorders, but it is also represented in most women as a primary form of disease. Pain associated with dysmenorrhea is caused by hypersecretion of prostaglandins and an increased uterine contractility. The primary dysmenorrhea is quite frequent in young women and remains with a good prognosis, even though it is associated with low quality of life. The secondary forms of dysmenorrhea are associated with endometriosis and adenomyosis and may represent the key symptom. The diagnosis is suspected on the basis of the clinical history and the physical examination and can be confirmed by ultrasound, which is very useful to exclude some secondary causes of dysmenorrhea, such as endometriosis and adenomyosis. The treatment options include non-steroidal anti-inflammatory drugs alone or combined with oral contraceptives or progestins.

## Introduction

Dysmenorrhea is defined as the presence of painful cramps of uterine origin that occur during menstruation and represents one of the most common causes of pelvic pain and menstrual disorder. The International Association for the Study of Pain defines pain as “an unpleasant sensory and emotional experience associated with actual or potential tissue damage, or described in terms of such damage” ^1^. In particular, chronic pelvic pain is located in the pelvic area and lasts for 6 months or longer ^2^.

The burden of dysmenorrhea is greater than any other gynecological complaint ^3^: dysmenorrhea is the leading cause of gynecological morbidity in women of reproductive age regardless of age, nationality, and economic status ^4– 7^. The effects extend beyond individual women to society, resulting annually in an important loss of productivity ^8, 9^. Thus, the World Health Organization estimated that dysmenorrhea is the most important cause of chronic pelvic pain ^10^.

The estimated prevalence of dysmenorrhea is high, although it varies widely, ranging from 45 to 93% of women of reproductive age ^3, 10^, and the highest rates are reported in adolescents ^11, 12^. Because it is accepted as a normal aspect of the menstrual cycle and therefore is tolerated, women do not report it ^13^ and do not seek medical care ^13, 14^. Some women (3 to 33%) have very severe pain, severe enough to render them incapacitated for 1 to 3 days each menstrual cycle, requiring absence from school or work ^15, 16^. Indeed, dysmenorrhea has a high impact on women’s lives, resulting in a restriction of daily activities ^17, 18^, a lower academic performance in adolescents ^19, 20^, and poor quality of sleep ^21^, and has negative effects on mood, causing anxiety and depression ^22^.

## Definition and pathogenesis

On the basis of pathophysiology, dysmenorrhea is classified as primary dysmenorrhea (menstrual pain without organic disease) or secondary dysmenorrhea (menstrual pain associated with underlying pelvic pathology) ^23^. The cause of primary dysmenorrhea is not well established. However, the responsible cause has been identified on the hyper-production of uterine prostaglandins, particularly of PGF _2a_ and PGF _2_, thus resulting in increased uterine tone and high-amplitude contractions ^24^. Women with dysmenorrhea have higher levels of prostaglandins, which are highest during the first two days of menses ^25^. Prostaglandin production is controlled by progesterone: when progesterone levels drop, immediately prior to menstruation, prostaglandin levels increase ^13, 24^. If the exposure of endometrium to luteal phase is crucial for the increased production of progesterone, dysmenorrhea occurs only with ovulatory cycles. This could explain why primary dysmenorrhea onset is shortly after menarche and why dysmenorrhea responds well to ovulatory inhibition. However, multiple other factors may play a role in the perception and the severity of pain, which does not depend only on endocrine factors ^26^.

The recurrent menstrual pain is associated with central sensitization, which is associated with structural and functional modification of the central nervous system ^24, 27^. Given that dysmenorrhea might led to important long-term consequences and may be increasing women’s susceptibility to others chronic pain conditions later in life, it is mandatory to treat menstrual pain in order to limit the noxious input into the central nervous system ^24^. The most common causes of secondary dysmenorrhea in young women are endometriosis and adenomyosis.

### Endometriosis

Endometriosis is characterized by the presence of endometrial tissue (glands and stroma) outside the uterine cavity and is the most common cause of secondary dysmenorrhea ^27, 28^. Pain symptoms negatively influence physical and psychological well-being of women with endometriosis. All forms of pain induce elevated sympathetic nervous system activity and this is considered a stressor, inducing changes in neuromediators, neuroendocrine, and hormonal secretions ^27, 29^.

Given that women with endometriosis wait before getting the right diagnosis ^30^, a great deal of effort has been made in recent years to try to find signs and symptoms that would help in making an earlier diagnosis. The early identification of these symptoms could help reduce the delay necessary for diagnosis ^15^ and enable the use of less invasive procedures ^31^. An early age onset of dysmenorrhea is considered a risk factor for endometriosis ^32^; other menstrual characteristics such as cycle length and menstrual bleeding duration and quantity are not related to the development of endometriosis. Parameters that may predict a later finding of deep infiltrating endometriosis are prolonged use of oral contraceptives (OCs) for treating primary dysmenorrhea, absenteeism from school during menstruation, and a positive family history of dysmenorrhea ^33^.

The endometriosis prevalence is higher in adolescents with chronic pelvic pain resistant to treatment with OC pills and non-steroidal anti-inflammatory drugs (NSAIDs) and in girls with dysmenorrhea ^34^. Therefore, severe dysmenorrhea that does not respond to medical therapy warrants further investigation such as by laparoscopy ^35^.

### Adenomyosis

Adenomyosis is defined as the presence of endometrial glands and stroma within the myometrium and is associated with dysmenorrhea and abnormal uterine bleeding (AUB). Adenomyosis is one of the most common causes of AUB ^36^. The diagnosis is usually confirmed through transvaginal ultrasonography and magnetic resonance imaging. Via specific ultrasonographic criteria by bidimensional and tridimensional transvaginal ultrasound (morphological uterus sonographic assessment) ^37^, the detection of adenomyosis features by imaging is accepted and the association with menstrual pain, heavy menstrual bleeding, and infertility may facilitate the diagnosis of adenomyosis ^38^. A 34% incidence of adenomyosis ultrasonographic features is found in young nulligravid women 18 to 30 years of age and is associated with dysmenorrhea ^39^.

## Risk factors

Heavy menstrual bleeding and longer menstrual bleeding duration are often associated with dysmenorrhea ^3, 20^. Childbearing is a very influential factor for the decrease of dysmenorrhea ^5^. Increasing age is also associated with less severe dysmenorrhea ^12^, although a longitudinal study found that the proportion of women with moderate to severe dysmenorrhea remained constant with increasing age ^5^.

The early onset of pain is associated with more severe pain ^3^, and a family history of dysmenorrhea is associated with a significantly higher prevalence of dysmenorrhea ^20^. Since anxiety and depression are often associated, dysmenorrhea may be part of a somatoform syndrome ^3^.

## Diagnosis

**Figure 1.**
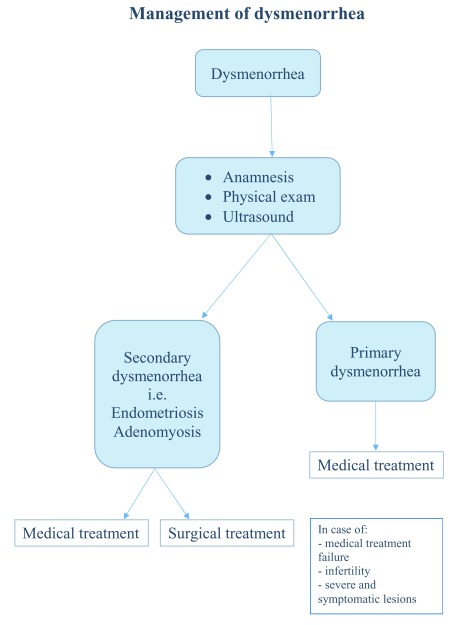
Flowchart for the management of patients with dysmenorrhea. Flowchart for the management of patients with dysmenorrhea.

A focused history and physical examination are usually sufficient for making a diagnosis of primary dysmenorrhea ^23, 26^. The onset of primary dysmenorrhea is usually 6 to 12 months after menarche. The typical pain is sharp and intermittent, is located in the suprapubic area, and develops within hours of the start of menstruation and peaks with maximum blood flow ^23^. The physical examination is completely normal, and the menstrual pain may be associated with systemic symptoms, such as nausea, vomiting, diarrhea, fatigue, fever, headache, and insomnia ^11, 16, 40^. There is no evidence for routine use of ultrasound in the evaluation of primary dysmenorrhea, although ultrasound is very useful in excluding the secondary causes of dysmenorrhea, such as endometriosis and adenomyosis ^26^ ( Figure 1).

Dysmenorrhea that occurs any time after menarche, that is associated with other gynecological symptoms such as dyspareunia, heavy menstrual bleeding, AUB, and infertility, and that does not respond to treatment with NSAIDs or OCs might be suspicious for secondary dysmenorrhea ^23, 24^. In particular, the analysis of menstrual bleeding abnormalities associated with dysmenorrhea might be helpful for the diagnosis of adenomyosis ( Figure 1).

## Treatment

The aim of the treatment for primary dysmenorrhea is pain relief.

### Non-steroidal anti-inflammatory drugs

NSAIDs are usually the first-line therapy for dysmenorrhea and should be tried for at least three menstrual periods ^41, 42^. If NSAIDs alone are not sufficient, OCs can be combined with it. NSAIDs are drugs that act by blocking prostaglandin production through the inhibition of cyclooxygenase, an enzyme responsible for formation of prostaglandins. Common NSAIDs (aspirin, naproxen, and ibuprofen) are very effective in reliving period pain ^43^. They make the menstrual cramps less severe and can prevent other symptoms such as nausea and diarrhea ^44^. NSAIDs reduce moderate to severe pain in women with primary dysmenorrhea ^23^. With the widespread availability of NSAIDs, the management of dysmenorrhea is mainly self-care ^13, 18^.

### Oral contraceptives

Contraceptive hormones act by suppressing ovulation and causing no endometrial proliferation ^13^. OCs bring almost immediate relief from symptoms associated with menstruation: heavy periods, painful periods, and irregular bleeding. In addition, OCs often are used as therapeutic drugs for women with symptomatic menorrhagia or endometriosis ^45, 46^.

The effectiveness of OC therapy for treating dysmenorrhea, regardless of the administration route (oral, transdermal, intravaginal, or intrauterine), has been shown ^12, 46– 51^. The use of OCs in a continuous fashion can be considered to treat primary dysmenorrhea, with two main advantages: the reduction of associated menstrual disorders and the improvement in women’s pain relief ^26^. However, limited evidence supports the use of OCs as a standard treatment ^23^.

The choice between the use of combined OCs and oral progesterone should be guided by the patient’s pain relief, the toleration of possible adverse effects especially linked to the frequency of breakthrough bleeding and weight gain, and the patient’s basal risk of venous thromboembolism ^52^.

### Progestins

Hormonal progestins-only treatment produces a benefit on menstrual pain, causing endometrial atrophy and inhibiting ovulation. Several long-acting reversible progestin contraceptives have been found to be effective treatments for primary dysmenorrhea. These include 52-mg (20 µg/day) levonorgestrel-releasing intaruterine system, the etonogestrel-releasing subdermal implant, and depot medroxyprogesterone ^53^.
